# Femoral osteotomy to improve range of motion in residual deformity of perthes disease: A case report

**DOI:** 10.1016/j.amsu.2020.04.031

**Published:** 2020-05-11

**Authors:** Shohei Matsubayashi, Ko Chiba, Ritsu Tsujimoto, Makoto Osaki, Akifusa Wada

**Affiliations:** a Department of Orthopedic Surgery, Graduate School of Biomedical Sciences, Nagasaki University, Japan; b Department of Orthopaedic Surgery, Saga Handicapped Children's Hospital, Japan

**Keywords:** Perthes disease, Residual deformity, 3DCT simulation, Valgus-flexion-internal rotation osteotomy, Case report, range of motion, (ROM), The Japanese Orthopaedic Association, (JOA), Computed tomography, (CT), three-dimensional CT, (3DCT), total hip arthroplasty, (THA), Systematic Utilitarian Procedure for Extremity Reconstruction, (SUPER)

## Abstract

**Introduction:**

The treatment strategies for residual deformity of Perthes disease are not established.

**Case presentation:**

This is a case report of a 15-year-old boy. He developed right Perthes disease (lateral pillar classification group B) when he was 10 years old and underwent varus femoral osteotomy of the right side. At 12 years of age, he developed left Perthes disease (lateral pillar classification group B) and underwent varus femoral osteotomy of the left side. Postoperatively, he was treated with partial weight bearing of the left leg with crutches. At 15 years, range of motion (ROM) of his left hip was markedly limited at 30° flexion, 10° abduction, 70° external rotation, and −20° internal rotation, and he was having difficulty maintaining a sitting position.

**Diagnosis:**

Stulberg group V was noted on plain radiography. Computed tomography (CT) showed collapse of the load-bearing part of the femoral head on the coronal plane, but the ball-shape was maintained in the posterior femoral head on sagittal and transverse sections.

**Intervensinos:**

Valgus-flexion-internal rotation osteotomy was performed to improve ROM.

**Outcomes:**

Left hip ROM improved to 90° flexion, 20° abduction, 50° external rotation, and 40° internal rotation immediately after the surgery. He was able to sit 10 months postoperatively but was left with a limp and limited ROM in the left hip at 60° flexion. Chondroplasty was performed during the plate removal surgery at 10 months postoperatively, which improved hip flexion to 100° immediately after the surgery. The patient was left with limited ROM of 60° flexion of the left hip at the final observation.

**Conclusion:**

Femoral osteotomy to improve ROM could be an option for residual deformity of Perthes disease.

## Highlights

• Treatment strategies for residual deformity of Perthes disease after skeletal maturity are not established.
• Soft tissues must also be altered to achieve ROM.
• Valgus-flexion-internal rotation osteotomy was performed as salvage operation for residual deformity of Perthes disease and improved ROM.

## 1 Introduction

The prognosis of late-onset Perthes disease, 9 years or older, is generally poor [1]. Hinge abduction may also occur in the late reossification or healed stages in late-onset Perthes disease to cause irreducibility. In these cases, treatment should aim for joint congruity rather than containment of the femoral head [2]. Various treatments have been reported for noncontainable Perthes hip, such as valgus osteotomy of the femur [2,3], shelf acetabuloplasty [4], chiari osteotomy [5], arthrodiastasis [6], and osteochondroplasty [7]. However, treatment strategies for residual deformity of Perthes disease are not established. We performed salvage operation to improve ROM in a patient with marked limited ROM resulting from residual deformity of Perthes disease and report herein.

Patient has provided informed consent for publication of the case.

All procedures performed in this study were in accordance with ethical stanndards of the Ethics Committee of Nagasaki University Graduate School of Biomedical Sciences (approval number:19041528). This study was conducted and reported in line with SCARE 2018 criteria [8].

## 2 Case presentation

Case: A 15-year-old boy.

Medical and family history: Nothing particular to note.

History of present illness: Pain in the right hip and right Perthes disease was detected at 10 years of age (classified as lateral pillar classification group B, Catterall group 3). Varus femoral osteotomy was performed on the right side.

**Fig. 1:**
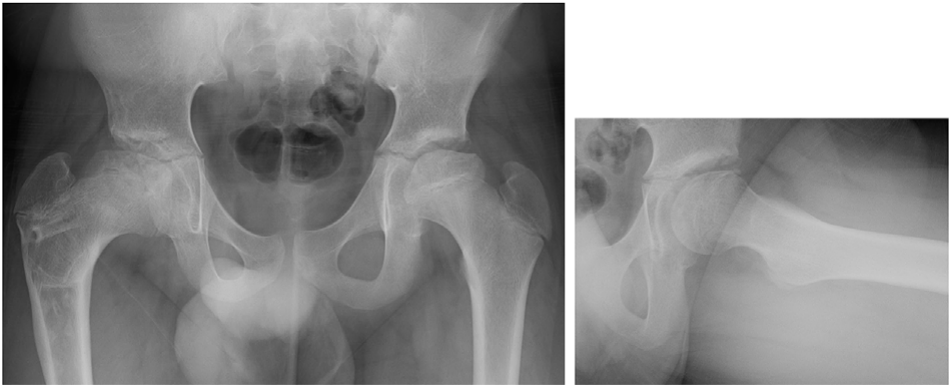
Initial stage, lateral pillar group B, Catterall group 2.

**Fig. 2:**
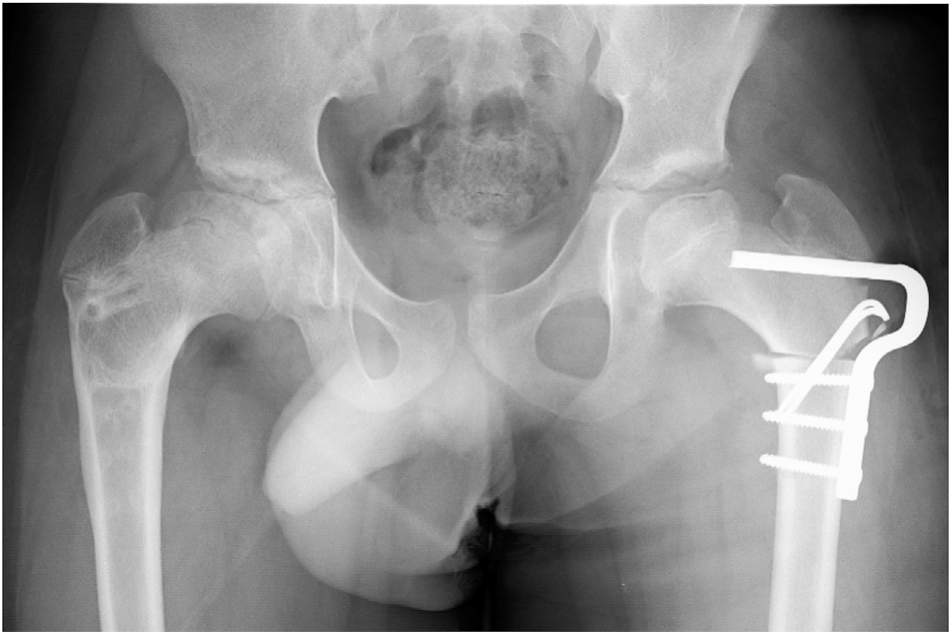
Varus femoral osteotomy was performed.

Left hip pain and left Perthes disease was detected at 12 years of age (classified as lateral pillar classification group B, Catterall group 2, Initial stage) (Fig. 1). Varus femoral osteotomy was also performed on the left side (Fig. 2).

The patient walked on crutches postoperatively to relieve load on the left leg. Limited ROM of the left hip was evident at 15 years of age. He limped and had difficulty sitting but did not have pain when he was referred to our department.

**Fig. 3:**
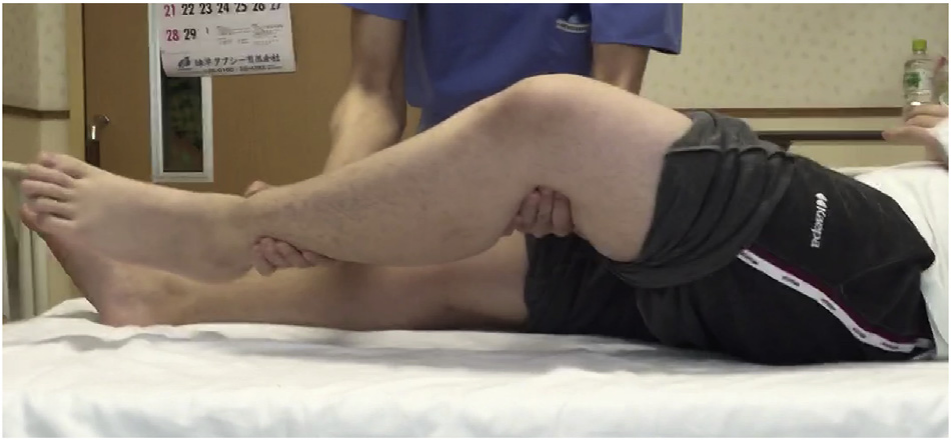
Positive Drehmann sign.

Physical findings: The patient's height was 165 cm, weight 75 kg, and body mass index 27.5. Limited ROM of the left hip: His ROM in the left hip was 30° flexion, 10° abduction, 70° external rotation, −20° internal rotation, and Drehmann sign-positive (Fig. 3). His hip score on The Japanese Orthopaedic Association (JOA) was 18 out of 100 points.

**Fig. 4:**
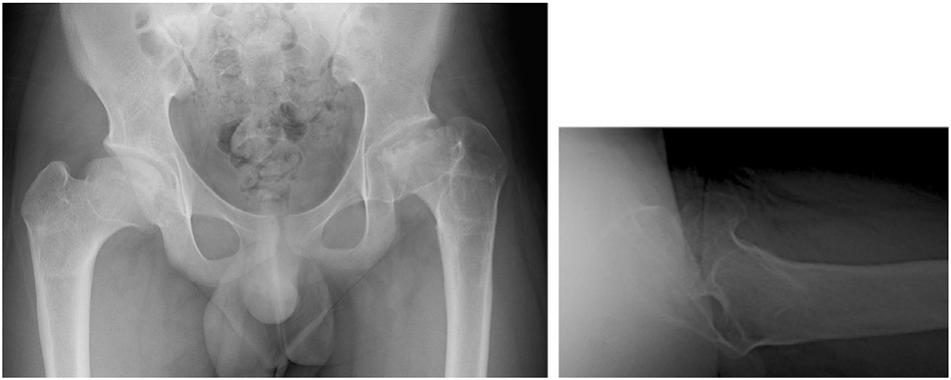
Stulberg group Ⅴ

**Fig. 5:**
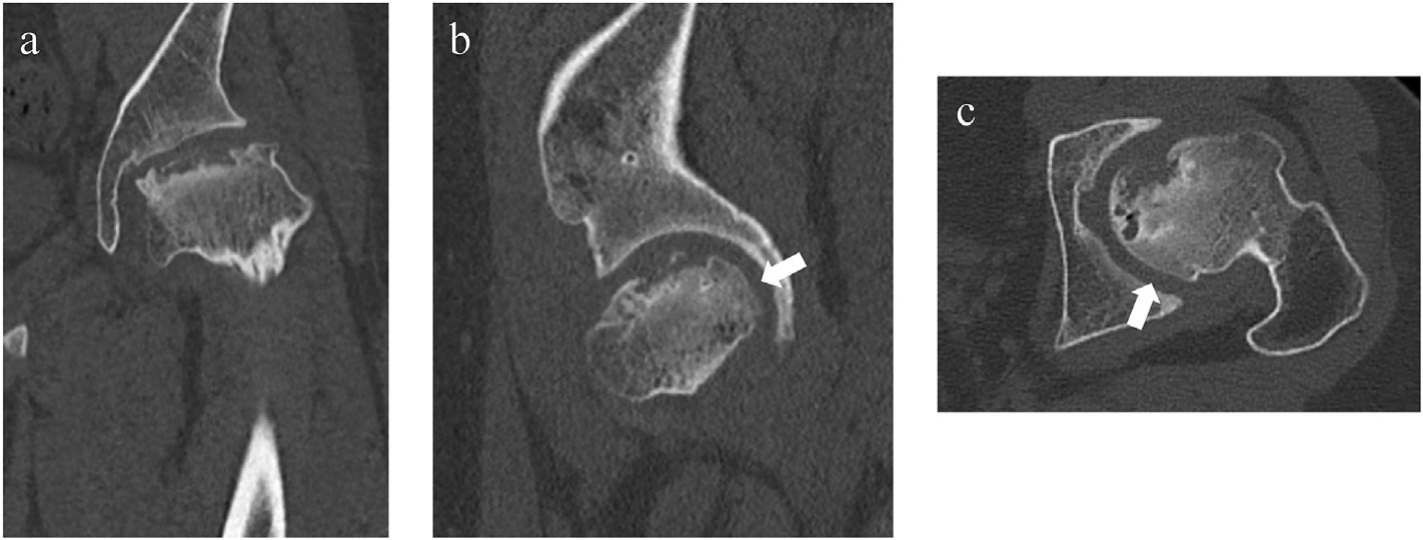
Computed tomography, a) Coronal view, b) Sagittal view, c) Axial view.

Imaging findings: Stulberg group V was noted on plain radiography (Fig. 4). Poor congruency of the joint surfaces at maximum abduction and adduction was also detected on functional image of plain radiography. Computed tomography (CT) showed collapse of the load-bearing part of the femoral head on the coronal plane (Fig. 5 a), but the ball-shape was maintained in the posterior femoral head on sagittal and transverse sections (Fig. 5b and c).

**Fig. 6:**
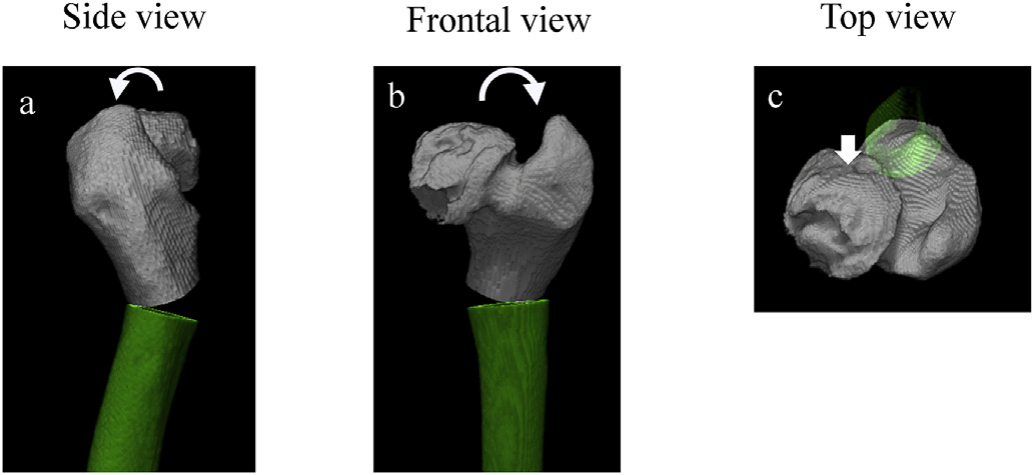
3DCT simulation, a) Side view, b) Frontal view, c) Top view.

Preoperative plan: Salvage operation was planned to improve ROM. 30° flexion osteotomy for the restricted flexion (Fig. 6 a), 30° internal rotation osteotomy for restricted internal rotation, and 20° valgus osteotomy (Fig. 6 b) for restricted abduction were planned. Anterior displacement of the collapsed part of the femoral head and displacement on the load-bearing surface of the posterior part with retained ball shape was identified on three-dimensional CT (3DCT) simulation (Fig. 6 c).

**Fig. 7:**
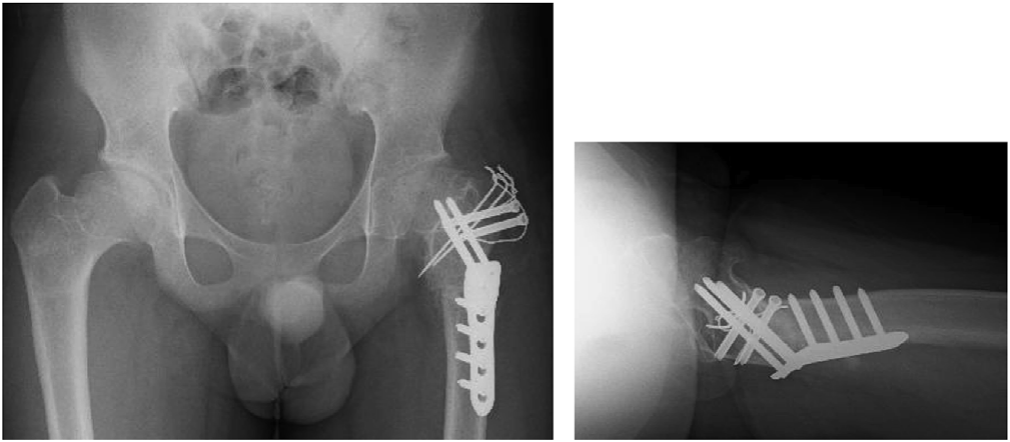
Valgus-flexion-internal rotation osteotomy was performed.

Operative findings: The adductor longus and gracilis muscles were completely cut through a medial approach in the lateral decubitus position. Next, 10 × 10-cm resection of the fascia lata was performed through a lateral approach. Twenty degrees valgus, 30° flexion and 30° internal rotation osteotomy and distal advancement of the greater trochanter were performed according to the preoperative plan (Fig. 7). The operation time was 4 hours and the bleeding volume was 126ml. The senior author (S.M), who had more than 20 years of experience, performed the operatiom.

Postoperative course: ROM improved from flexion 30°to90°, abduction 10°to20°, external rotaion 70° to 50°and internal rotation −20° to 40° immediately after the surgery. The limp persisted, but walking was possible at 10 months postoperatively. Plain radiography showed that collapse of the femoral head's load-bearing surface was not progressing. However, the limited ROM of the left hip remained at 60° flexion.

**Fig. 8:**
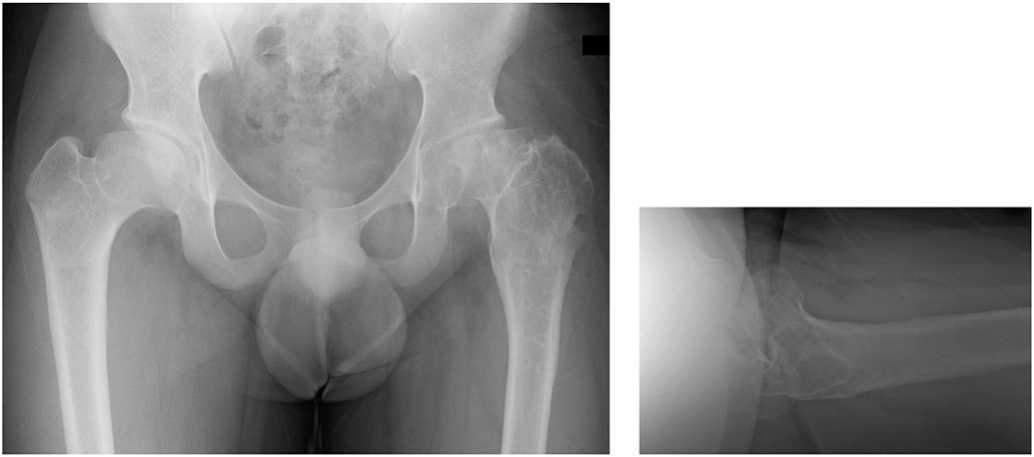
Final follow-up.

**Fig. 9:**
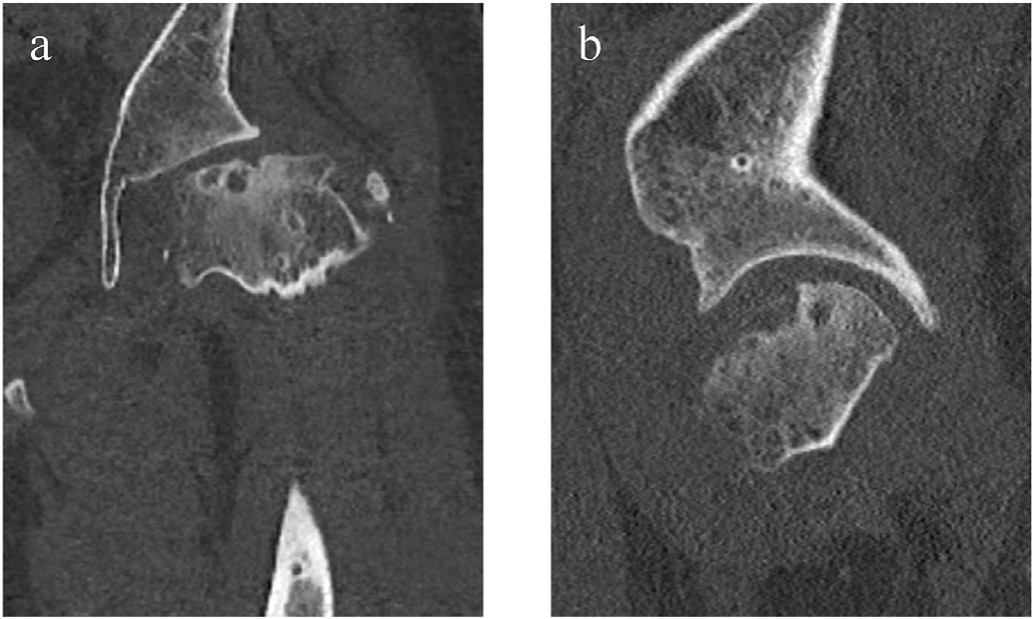
Computed tomography, a) Coronal view, b) Sagittal view at final follow-up.

Chondroplasty was performed during plate removal surgery at 10 months postoperatively. Hip flexion improved to 100° immediately after the surgery, but a limited hip ROM of 60° flexion, 20° abduction, 60° external rotation and 15° internal rotation remained at the final observation. However, the JOA hip score improved to 64 out of 100 points. Plain radiography showed gradual remodelling of the femoral head's load-bearing surface (Fig. 8). In addition, CT showed gradual remodelling of the femoral head's load-bearing surgace (Fig. 9a and b).

## 3 Discussion

Although various treatments for noncontainable Perthes hip have been reported [[2], [3], [4], [5], [6], [7]], treatment strategies for residual deformity of Perthes disease after skeletal maturity are not established. Osteotomy for hip arthrosis in young patients requires ≥80° flexion to prevent postoperative spontaneous ankylosis [9]. Perhaps total hip arthroplasty (THA) should be considered immediately for patients with only 30° flexion. However, THA should be cautiously indicated, considering the patient's young age and because high revision rate was reported THA for sequelae of Perthes disease [10].

Choi et al. performed valgus osteotomy for a noncontainable Perthes hip to obtain congruency with added flexion-internal rotation osteotomy [2]. The surgery we performed in this patient was similar, but the concept was different. A functional image of plain radiography showed poor congruency of the joint surface at the maximum abduction and adduction. CT showed collapse of the femoral head's load-bearing part on the coronal plane. Thus, the goal of the surgery was changed from achieving congruency to restoring ROM. Valgus osteotomy was performed for the limited abduction, flexion osteotomy was performed for the limited flexion, and internal rotation osteotomy for the limited internal rotation. Furthermore, a 3DCT simulation showed anterior displacement of the collapsed site of the femoral head, and displacement of the posterior part with maintained ball shape on the load-bearing surface.

Soft tissues must also be altered to achieve ROM. Yoo et al. reported the importance of releasing soft tissue contractures in noncontainable Perthes hip [3]. Complete cut of the adductor longus and gracilis muscles was also performed via a medial approach on this patient. And the iliopsoas tendon was cut from the lesser trochanter at the osteotomy. The vastus lateralis was not sutured after the osteotomy. Furthermore 10 × 10-cm total resection of the fascia lata via the lateral approach was performed to reach the femoral bone. Because we believe the fascia lata is greatly involved in hip joint contractures. Paley et al. also resected the fascia lata in the Systematic Utilitarian Procedure for Extremity Reconstruction (SUPER) hip procedure performed to treat congenital femoral deficiency [11].

Hip flexion improved from 30° preoperatively to 60° at final observation. This made it easier for the patient to get sitting position. The flexion osteotomy was fully effective. To further improve the flexion angle, it would be better to have a larger flexion osteotomy angle at the surgery. However, if the flexion osteotomy angle is increased, the extension anlgle of the hip joint may be limited and walking may be restricted. The angle of flexion osteotomy at the surgery would have to be considered on an individual case basis.

## 4 Patient consent

Written informed consent was obtained from patient's mother for publication of this case report and accompanying images. A copy of the written consent is available review by Editor-in-Chief of this journal on request.

## 5 Provenance and peer review

Not commissioned, externally peer reviewed.

There is a limitation of this study though: the follow-up period is short. So it is necessary to continue monitoring the patient's condition.

## 6 Conclusion

Valgus-flexion-internal rotation osteotomy was performed as salvage operation for residual deformity of Perthes disease and improved ROM.

## Guarantor

The Guarantor is the one or more people who accept full responsibility for the work and/or the conduct of the study, had access to the data, and controlled the decision to publish.

## Author contribution

Shohei Matsubayashi, MD., PhD: Corresponding Author.

Ko Chiba, MD., PhD: data collection.

Ritsu Tsujimoto, MD., PhD: data analysis.

Makoto Osaki, MD., PhD: interpretation.

Akifusa Wada, MD., PhD: study concept.

## Ethical approval

All procedures performed in this study were in accordance with ethical stanndards of the Ethics Committee of Nagasaki University Graduate School of Biomedical Sciences (approval number:19041528)

## Declaration of competing interest

The authors declare that they have no known competing interests or personal relationships that could have appeared to influence the work reported in this paper.
